# Routine Pediatric Surgical Emergencies: Incidence, Morbidity, and Mortality During the 1st 8000 Days of Life—A Narrative Review

**DOI:** 10.1007/s00268-023-07097-z

**Published:** 2023-06-21

**Authors:** Alizeh Abbas, Ruth Laverde, Ava Yap, Caroline Q. Stephens, Lubna Samad, Justina O. Seyi-Olajide, Emmanuel A. Ameh, Doruk Ozgediz, Kokila Lakhoo, Stephen W. Bickler, John G. Meara, Donald Bundy, Dean T. Jamison, Greg Klazura, Alicia Sykes, Godfrey Sama Philipo, On behalf of GICS

**Affiliations:** 1 Department of Surgery, University of Alabama at Birmingham, Birmingham, AL USA; 2 Department of Surgery, University of California San Francisco, San Francisco, CA USA; 3 Global Surgery Programs, Interactive Research & Development, Karachi, Pakistan; 4 Pediatric Surgical Unit, Lagos University Teaching Hospital, Lagos, Nigeria; 5 Division of Pediatric Surgery, Department of Surgery, National Hospital, Abuja, Nigeria; 6 Department of Pediatric Surgery, University of Oxford and Oxford University Hospitals, Oxford, UK; 7 Division of Pediatric Surgery, Department of Surgery, University of California San Diego School of Medicine, 9500 Gilman Drive #0739, La Jolla, San Diego, CA 92093-0739 USA; 8 Program in Global Surgery and Social Change, Harvard Medical School, Boston, MA USA; 9 Global Research Consortium for School Health and Nutrition, London School of Hygiene and Tropical Medicine, London, UK; 10 Institute for Global Health Sciences, University of California, San Francisco, San Francisco, CA USA; 11 Loyola University Medical Center, Chicago, IL USA; 12 Naval Medical Center San Diego, San Diego, CA USA; 13 Muhimbili University of Health and Allied Sciences, Dar es Salaam, Tanzania

## Abstract

**Background:**

Many potentially treatable non-congenital and non-traumatic surgical conditions can occur during the first 8000 days of life and an estimated 85% of children in low- and middle-income countries (LMICs) will develop one before 15 years old. This review summarizes the common routine surgical emergencies in children from LMICs and their effects on morbidity and mortality.

**Methods:**

A narrative review was undertaken to assess the epidemiology, treatment, and outcomes of common surgical emergencies that present within the first 8000 days (or 21.9 years) of life in LMICs. Available data on pediatric surgical emergency care in LMICs were aggregated.

**Results:**

Outside of trauma, acute appendicitis, ileal perforation secondary to typhoid fever, and intestinal obstruction from intussusception and hernias continue to be the most common abdominal emergencies among children in LMICs. Musculoskeletal infections also contribute significantly to the surgical burden in children. These “neglected” conditions disproportionally affect children in LMICs and are due to delays in seeking care leading to late presentation and preventable complications. Pediatric surgical emergencies also necessitate heavy resource utilization in LMICs, where healthcare systems are already under strain.

**Conclusions:**

Delays in care and resource limitations in LMIC healthcare systems are key contributors to the complicated and emergent presentation of pediatric surgical disease. Timely access to surgery can not only prevent long-term impairments but also preserve the impact of public health interventions and decrease costs in the overall healthcare system.

## Introduction

Most global child health efforts have concentrated on the first 1000 days of life, from conception to the second birthday. Yet, according to the *Disease Control Priorities* 3rd Edition volume on “Child and Adolescent Health and Development” it takes 8000 days (or 21.9 years) to develop into an adult from infancy [1]. This is of particular importance in low- and middle-income countries (LMICs), as an estimated 85% of children in these settings will develop a potentially treatable surgical condition before the age of 15 [2]. However, data on emergency surgical care among children in LMICs remains limited [3]. This is concerning since the majority of the 1.7 billion children who do not have access to surgical treatment live in LMICs, where the proportion with no surgical access can be as high as 92–98% [4].

Acute appendicitis and associated complications, ileal perforation secondary to typhoid fever, intussusception, and incarcerated hernias continue to be the most common abdominal emergencies among children in LMICs and represent a considerable proportion of the burden of disease globally, comparable to congenital anomalies [5, 6]. Emergency pediatric surgical procedures, especially those that treat infections and trauma, utilize a considerable portion of healthcare resources in LMICs, accounting for more than half of the surgical capacity or admissions to pediatric surgery services [7–10].

Despite the substantial surgical burden of disease in children and the resources required to address this, discussion on the role of surgery in improving the survival and quality of life in children is frequently absent from the public health agenda [4]. Furthermore, numerous children’s emergency surgical pathologies such typhoid intestinal perforation and osteomyelitis preferentially affect LMIC populations [11, 12] and such neglected surgical diseases in LMICs have been identified as priority investments. Alarmingly, adjusted mortality in children following emergency abdominal surgery may be 5–7 times greater in LMICs compared to HICs [5], providing strong ethical reasons for prioritizing interventions to address these gaps.

This report will review the non-traumatic emergency surgical procedures occurring in LMICs. Traumatic surgical conditions and congenital anomalies requiring urgent surgery will be highlighted in other reports of this series of global pediatric surgery. We aim to cover the burden, morbidity, and mortality of the existing priorities for emergency general surgical diseases including common abdominal and musculoskeletal conditions affecting children in LMICs. As a narrative review, relevant literature was searched using key words including LMIC, pediatric surgical emergencies, appendicitis, intestinal perforation, intussusception, hernia repair, children’s surgical emergencies, emergency laparotomy. We excluded articles that included only adult patients and focused solely on high-income countries (HICs). For each article we identified: year, setting/country, conditions included, exact age range, incidence, prevalence, complications/morbidity, and associated mortality rates, and any discussion of cost implications if included.

## Intra-abdominal infections

Abdominal sepsis due to intestinal perforation is a deadly complication following advanced intrabdominal infections such as typhoid fever or perforated appendicitis and requires emergency surgical intervention. These infections are most common in school age and adolescent children and may often go unrecognized or be undertreated at an early stage. [13, 14]. This delay in care results in prolonged hospitalizations, additional surgeries, and preventable post-operative complications, further contributing to pediatric morbidity and mortality [15, 16].

### Typhoid fever

Typhoid fever is an infection caused by *Salmonella typhi* transmitted through the fecal–oral route, typically from contaminated food or poor water sanitation. Perforation due to typhoid fever or typhoid ileal perforation is a significant issue in LMICs and almost absent in HICs. The annual incidence of typhoid is estimated at 21 million cases, with an overall perforation rate of about 10% that increases with age, reaching a high of 30% by age 12 years [17]. Mortality ranges from 12% in surgically equipped facilities to 100% in areas without adequate surgical access [10, 14, 18, 19]. For those children who do survive, the majority experience one or more complications, including surgical site infections, dehiscence, and enterocutaneous fistulae causing significant morbidity [16, 18]. Typhoid fever can also cause other complications that may require surgery, including cholecystitis, osteomyelitis, and abscesses. Further work on this condition has emphasized the consequences of delayed diagnosis due to multifactorial causes and resource shortages in surgical systems. In addition, reviews have also emphasized the need for prevention of this condition through distribution of the typhoid vaccine and improved sanitation [20]. Routine vaccination against multi-drug resistant typhoid show promise in reducing disease burden globally, potentially averting 42.5 million cases [21]. Table 1 summarizes recent literature on typhoid intestinal perforation and expands on a prior scoping review.

**Table 1:** Most common complications and mortality rates due to typhoid intestinal perforation in LMIC as reported in the literature

Country	Study years	Author	Top three complications	Mortality rate n (%)
*LMIC other than Nigeria*				
Abidjan, Ivory Coast	1990–2000	Kouame et al.	Surgical Site Infections (SSI), Enterocutaneous Fistula (ECF), Incisional hernia	3 (6)
Kumasi, Ghana	1995–1997	Abantanga et al.	SSI, Wound dehiscence, chest infection	15 (12.4)
Bangui, Central African Republic	1997–1998	Bobossi Se´re´ngbe´ et al.	SSI, incisional hernia, evisceration	9 (29)
Kumasi, Ghana	2001–2005	Abantanga et al.	Sepsis, postoperative continuing peritonitis, wound infection and wound dehiscence	82 (12.6)
Bamako, Mali	2005–2010	Coulibaly et al.	(Could not find full text article in English)	16 (15.2)
Tanzania*	2006—2011	Chalya et al.	SSI, chest infection, septic shock	24 (23.1)
Zinder, Niger	2013–2015	Adamou et al.	(Could not find full text article in English)	22 (14.4)
Pakistan	2016–2019	Azhar et al.	SSI, intra-abdominal collection, burst abdomen	12 (12.37)
*Nigeria*				
Ilorin, Nigeria	1984–1999	Rahman et a.l	SSI, ECF	25 (23.6)
Ibadan, Nigeria	1985–2000	Irabor	SSI, wound dehiscence, incisional hernia	39 (21.3)
Zaria, Nigeria	1987–1996	Ameh	SSI, chest infection, wound dehiscence	25 (39)
Benin City, Nigeria	1993–2007	Osifo et al.	ECF, SSI, evisceration	9 (75)
Ile-Ife, Nigeria	1994–2004	Usang et al.	SSI, wound dehiscence, intra-abdominal abscess, and ECF (equal percentage for last two)	9 (23.7)
Enugu, Nigeria	1995–2004	Ekenze et al.	SSI, chest infection, re-perforation	17 (19.1)
Jos, Nigeria	1996–2005	Uba et al.	SSI, wound dehiscence, chest infection	42 (22.8)
Enugu, Nigeria	2001–2006	Ekenze et al.	SSI, chest infection, wound dehiscence	21 (25.3)
Ilorin, Nigeria	2002–2009	Nasir et al.	ECF, wound dehiscence, evisceration	16 (10.4)
Azare, Nigeria	2004–2008	Nuhu et al.	SSI, re-perforation, wound dehiscence	13 (28.3)
Ife, Nigeria	2005–2013	Talabi et al.	SSI, wound dehiscence, evisceration	9 (20)
Calabar, Nigeria	2006–2015	Usang et al.	SSI, chest infection, ECF	4 (8.2)
Kano, Nigeria	2007–2012	Ibrahim et al.	SSI, ECF, evisceration	42 (4.6)
Enugu, Nigeria	2008–2009	Ekenze et al.	SSI, wound dehiscence, incisional hernia	3 (13.6)
Ado-Ekiti, Nigeria	2008–2010	Adegoke et al.	(Not mentioned)	6 (12.8)
Kano, Nigeria	2009–2013	Anyanwu et al.	SSI, wound dehiscence, evisceration	14 (10.9)
Ibadan, Nigeria	2010–2017	Ajao et al.	(Complications after surgery for typhoid ileal perforation not stated clearly as the paper focused on indications of bowel resection)	1 (11.1)
Aba, Nigeria	2016–2018	Ekpemo et al.	SSI, chest infection, intra-abdominal abscess	5 (8.3)
Nigeria	2014 –2018	Chukwubuike	SSI, chest infection, ECF	3 (6.8)

Many details of this table have been adapted from Birkhold et al. [18] study and then updated with additional studies and details.

^*^Patient population not limited to pediatric age group

### Acute appendicitis

Acute appendicitis is caused by inflammation of the appendix, typically due to bacterial build up in the appendiceal lumen obstructed by a fecalith, or less commonly by neoplasm or lymphadenopathy. The duration of inflammation is directly proportional to the severity and extent of the infection, meaning early diagnosis and treatment is paramount. Complicated or perforated appendicitis can often be avoided with timely treatment and surgical intervention. According to available data derived from mostly single-institution retrospective chart reviews from hospitals in the Gambia, Uganda, and Nigeria, appendicitis was the diagnosis for up to 17.1% of surgeries [6, 22, 23]. Reported mortality rate in LMICs can reach as high as 3.5% [24, 25]**.** Due to delays in presentation, complicated appendicitis is more common in LMICs, composing up 67–85% of all presenting appendicitis cases in some studies [14, 26]. Perforation rates range from 40 to 60% in LMICs [24, 27, 28]. Perforation may also lead to further complications such as abscesses, fistulas, and ultimately abdominal sepsis if left inadequately treated. Treatment of these complications are generally more invasive and may require bowel resection and multiple procedures, which portend a higher risk of surgical site infections and other postoperative complications. Table 2 summarizes characteristics of recent literature on abdominal emergencies in children in LMICs.

**Table 2:** Characteristics of articles on abdominal surgical emergencies among children in LMIC

Country	Study years	Author	Condition	Age	Incidence	Prevalence	Complications/morbidity	Mortality	Cost implications
*Original*									
Nigeria	2014–2017	Seyi-Olajide et al.	Typhoid intestinal perforation	2–15 years	✔		✔	✔	✔
Nigeria	1996 –2005	Uba et al.	Typhoid intestinal perforation	4–15 years	✔		✔	✔	
Pakistan	2016–2019	Azhar et al.	Typhoid intestinal perforation	3–12 years	✔		✔	✔	
Ghana	2001 –2005	Abantanga et al.	Typhoid intestinal perforation, appendicitis, abdominal trauma, intestinal obstruction, irreducible hernia, gallbladder disease	1–14 years	✔	✔	✔	✔	
Tanzania	2006 –2011	Chalya et al.	Typhoid intestinal perforation	8–76 years	✔	✔	✔	✔	
Nigeria	1993–2007	David Osifo et al.	Typhoid ileal perforation	5–13 years	✔		✔	✔	
Nigeria	2014 –2018	Chukwubuike	Typhoid ileal perforation and ileal hemorrhage	6–14 years	✔		✔	✔	
*Review*									
Multiple		Jiang et al.	Intussusception	< 18 years	✔			✔	
Nigeria, Mali, Ghana, Niger, Ivory Coast, Central African Republic	1995–2019	Birkhold et al.	Typhoid Intestinal Perforation	2 months – 15 years			✔	✔	

## Intestinal obstruction

Indications for bowel resections secondary to intestinal obstruction in children in LMICs are mainly from acquired and preventable conditions, such as intestinal obstructions caused by intussusception and incarcerated hernias [5, 29].

### Intussusception

Intussusception is a condition in which one segment of intestine “telescopes” inside another, leading to intestinal obstruction. If not reduced in a timely fashion, swelling, inflammation, and ultimate perforation and abdominal sepsis ensues. Due to late presentation of intussusception in most African countries, the treatment has been mostly surgical [30]. Infants older than 4 weeks old and toddlers are most affected, but it may appear at any age. Intussusception is a leading cause of bowel obstruction and bowel resection in infants, however there is a paucity of demographic data in LMIC surrounding this condition [14]. The estimated annual incidence of childhood intussusception in Africa is 72 per 100,000 children, while reported cases in India, Vietnam, and China range from 2 to 54 per 100,000 to 200–800 per 100,000 per year. [30, 31]. However, these retrospective hospital-based studies probably underestimate the incidence of intussusception as they do not account for patients who may have died before reaching the hospital, presented to other facilities within the same region, or were treated with alternative diagnoses [30].

In Africa, the intestinal resection rates in patients presenting after more than 48 h ranged from 60 to 100% while for patients presenting within 48 h the corresponding value was 12.4% [30]. Some authors have used a 24 h cut-off to study outcomes; children who presented after 24 h of onset of symptoms had a higher incidence of bowel complications, a greater risk of failed operative reduction, and a higher chance of bowel resection at time of operation [14, 30]. At a teaching hospital in Ghana, more than 35% of children with intussusception underwent bowel resection for gangrenous bowel which was likely due to delayed presentation [14]. Late presentation leading to delayed surgery has also contributed to higher rates of post-operative complications such as septicemia, hemorrhage, and abscess formation [30].

Limited access to timely pediatric surgical and radiological expertise contributes to delay in diagnosis and surgical interventions [30]. In the absence of imaging such as ultrasound or plain film X-ray, definitive diagnosis can only be made using laparotomy [30, 32] Even without access to fluoroscope and/or ultrasound facilities, air enema reduction of intussusception can be attempted under anesthesia, with greater than 50% success rate if attempted early in the course of disease manifestation[17]. However, due to late presentation and lack of ultrasound or radiological services, especially outside normal work hours, pneumatic or hydrostatic reduction cannot be conducted, thus leaving surgical management as the only viable management option [30, 32]. This paradigm leads to a higher morbidity and mortality from intussusception in Africa than in other regions of the world [33]. Greater investment in rapid diagnosis, surgical services, and radiologic technology is needed to address this condition and reduce the likelihood of children undergoing bowel resection [30, 34].

### Incarcerated hernias

Abdominal wall hernias are another common cause of intestinal obstruction in LMICs, as loops of intestine can become trapped, incarcerated, and subsequently strangulated within these abdominal wall defects.

Umbilical hernias are especially common in Africa. Umbilical hernias can occur in 91% of children 5 years or younger and 46% of children who are 10–15-year-old [35]. In the United States, umbilical hernias occur in 40–60% of infants and only 4% remain present after the age of 5, while the remaining spontaneously close [36]. In general, umbilical hernias should be repaired by 5 years of age if the defect does not obliterate spontaneously. In single center studies, incarceration from an umbilical hernia can occur in 37–44% of patients presenting to the hospital with symptoms [37, 38].

In Africa, inguinal hernias are present in 1–5% of children overall and in 35% of premature babies [17]. In the United States, the condition has a similar prevalence of 1–5% in children overall and a lower prevalence at 9–11% in premature infants [39]. Similar incidence rates exist in Asia, where inguinal hernias occur in 6.6% of males and 0.7% of females in a nationwide Taiwanese population study [40]. Given the heightened risk of incarceration, early elective herniotomy is the treatment of choice after diagnosis of a reducible inguinal hernia.

Elective herniotomy is usually a short outpatient case and is extremely cost-effective [41]. However, surgical backlog or compromised access to surgical care frequently prevents timely repair of these defects, which leads to a high incidence of incarcerated and strangulated hernias in LMICs [38, 42, 43]. In a Nigerian study, obstructed hernias made up 14.8% of all abdominal surgical emergencies, ranking third after typhoid ileal perforation and intussusception. Inadequate surgical capacity to accommodate elective cases prevents early surgical correction of this common condition, leading to preventable morbidity that necessitates emergent repair.

## Musculoskeletal infections

Musculoskeletal infections make up a significant burden of disease in LMICs compared to HICs, straining already limited health care services, but these have been neglected as a public health priority. Joint and bone infections, such as acute septic arthritis and osteomyelitis, affect up to 12 million children, disproportionately affecting school age children [29, 44–46]. When these infections go unaddressed, children can often require multiple surgeries to control infection and often suffer limb loss, resulting in lifelong disability.

### Septic arthritis

Septic arthritis is a joint infection that is caused by hematogenous or contiguous spread of bacteria, and sometimes due to direct inoculation. The incidence of septic arthritis is higher in younger children [47]. Due to the varied epidemiology that mimic acute bacterial septic arthritis such as tuberculosis or human immunodeficiency virus (HIV) associated arthropathy, across LMICs, it can be challenging to clinically distinguish a bacterial infection from other causes of an inflamed joint [48]. Moreover, the clinical presentation can manifest as less acute in immunocompromised children, such as those who are malnourished or HIV infected, and radiological changes are not apparent immediately. The invariable lack of access to microbiology services can lead to delayed diagnosis and resultant chronic infection, that in turn lead to physical limitations, chronic sinus formation and subsequent superinfection [49]. In addition, osteomyelitis can develop if the infection spreads into the adjacent bone.

### Osteomyelitis

Osteomyelitis is a bone infection that often results from direct inoculation into bone or contiguous or hematogenous spread of an infection from elsewhere in the body. Acute hematogenous osteomyelitis is the most common form of osteomyelitis in school age children but can happen at any age. It is often associated with the need for invasive diagnostic, surgical procedures, and prolonged course of antimicrobial treatment. [9, 50]. Furthermore, there is an association of osteomyelitis with sickle cell disease, which has a higher prevalence in LMICs [51]. Studies from LMICs show that patients with osteomyelitis may be older than most surgical patients on admission and experienced a significantly longer average length of stay [50, 52, 53]. Delayed and/or inadequate treatment of acute osteomyelitis in LMIC settings results in 60–80% of affected children presenting in the chronic phase [44, 54, 55], leading to more complex and multi-stage management [54]. Delayed treatment of open bone fractures can also contribute to osteomyelitis in up to a quarter of patients [56, 57]. One review demonstrated that early administration of antibiotics is the most important factor in prevention, but timely intervention does not consistently occur in LMICs [58]. Lack of imaging and microbiology also pose added challenges. A study estimates that 27% of patients with osteomyelitis require both multiple admissions and surgeries before treatment is successful [46]. In low-income countries, eradication of chronic osteomyelitis after treatment is never assured, and many patients have recurring infections and ultimately may require amputation of the affected limb [59].

## Economic and societal impact

Even when children survive a surgical emergency, chronic disability due to untreated, partially treated, or unsuccessful surgical treatment may lead to social marginalization, school disengagement, and undue burden on caregivers [60]. Long hospitalizations from typhoid infection or chronic musculoskeletal infections can also deprive a child from attending school and interrupt education. Permanent cure from osteomyelitis after treatment is uncertain, and many patients with recurring infections suffer serious consequences in psychological state, dependence on others, and financial difficulties for the family [59]. Economic burden is especially common after emergency surgeries, which are difficult to financially prepare for, as a study in Nigeria found that 100% of patients who presented with typhoid intestinal perforation incurred catastrophic healthcare expenditure [61]. In addition to person-level economic burden, chronic conditions can impact regional economy. In a study conducted at a university hospital in South Africa, delayed diagnosis and indefinite treatment contributed significantly in the estimated £2 million spent in the management of appendicitis [24, 25].

## Delays in care

In all the conditions discussed above, delays in care are a major contributor to the preventable morbidity and mortality in LMICs [6]. For example, late presentation of intussusception is associated with a higher incidence of bowel complications and greater risk of failed operative reduction, need for bowel resection, and increased risk of mortality [62]. Likewise, acute appendicitis is a time sensitive pathology with worse outcomes secondary to delay [25, 63]. Patients with appendicitis who present with longer duration of symptoms have a higher rate of perforation [24]. As a result, patients commonly present with advanced surgical disease, such as perforated appendicitis or osteomyelitis secondary to improper treatment or delayed treatment of fractures. In addition, patients may present with medical conditions that would not initially require surgery but have progressed, such as osteomyelitis secondary to septic arthritis [2, 64].

Types of delay may be three-fold: in the decision to seek care, presentation to a care facility, and adequate treatment [65]. In the first delay, surgical diseases can originate from delay in diagnosis or misdiagnosis of medical conditions, resulting in a large hidden mortality as some preventable surgical deaths are misclassified as medical or unavertable. In the second delay, patients who recognize the need for surgery may not be able to access a capable surgeon due to the concentrated density of pediatric surgical specialist in major cities across LMICs. Therefore, access to appropriate surgical services can be limited to major central hospitals. Pre-hospital factors such as distance, time, or cost needed to travel may prevent children from receiving prompt care or persuade parents to first consult local traditional healers [66]. In the third delay, inadequate treatment can stem from the scarcity of trained providers and limited resources even once patients present to the hospital. Long wait times from surgical back log in addition to limited operating room space also contribute to delayed surgical interventions that turn elective surgical conditions in emergent ones. Thus, the missed opportunities to intervene convert conditions that are correctable into ones with a higher likelihood for complications and mortality [67].

## Future directions in health systems strengthening

Our review emphasizes the urgent need to strengthen surgical systems and improve access to trained personnel catering to the treatment of these surgical diseases. The WHO recently recommended "emergency and operative services" as a priority, stressing the importance of primary care, communication, transportation, and referral processes in connecting emergency, critical, surgical, and anesthetic services to communities [68]. Capacity failures in primary care and social services may result in greater need for emergency, critical care, and surgical services, leading to delays in the provision of needed life-saving treatment. Similarly, capacity failures in surgical systems may result in disrupted primary care delivery and poor outcomes. Therefore, the WHO emphasizes the need for universal access to emergency surgical care, as well as financial risk protection within a health system.

Greater investment in surgical care in rural and district hospitals is sorely needed to adequate address surgical needs in a timely manner. Additionally, as pediatric surgical capacity is primarily located in major cities, efforts to train general surgeons to treat emergent diseases in children is critical. Educating and training of non-specialized medical providers in the treatment of pediatric surgical diseases may be a viable approach in some settings. Successful examples of this model includes the Pediatric Emergency Surgical Care course that is held at district hospitals in Uganda [69] and the Provider’s Course for Children’s Surgical Care in secondary hospitals, which is a joint venture between the charity Global Initiative for Children’s Surgery (GICS), the Royal College of Surgeons in England and the Christian Medical College in Vellore, India [70]. GICS is also participating in the creation of educational tools for frontline healthcare providers at rural facilities through the WHO Academy, an open-access, online platform that would be open to all learners [71].

Future directions in surgical capacity building for children should therefore aim to bolster education of primary providers and general surgeons on recognition of urgent pediatric surgical disease and appropriate resuscitation, initial treatment, and timely referral. Relatedly, to ensure competency and quality in care, the WHO recommends establishing, as appropriate, regulation and certification mechanisms for all healthcare workers.

The Optimal Resources For Children’s Surgical Care (ORECS) document developed by the Global Initiative for Children’s Surgery provides comprehensive guidelines for strengthening surgical systems with a focus on surgical infrastructure and capital for high-burden conditions affecting children in low-resource settings. The OReCS guidelines provides a template that can be used by both policy makers and hospital leadership to streamline surgical care provision for children, especially at the first (or district) level of care.

## Conclusion

Children comprise up to half of the LMIC population, and surgical emergencies in this age group contribute significantly to the surgical disease burden. Overall, this review underscores not only the high mortality and morbidity in LMICs from common pediatric surgical emergencies such as bowel perforation, bowel obstructions, and musculoskeletal infection, but also the heterogeneity among LMICs. Children in LMICs are disproportionately impacted, suggesting that delayed or inadequate care are key contributors to complicated and emergent presentations, thus placing a substantial burden on already stretched healthcare resources. Timely access to surgery can not only prevent long term impairments from injuries, infections, and acute exacerbations of non-communicable disease, but also preserve the impact of other public health interventions and decrease cost in the overall health care system.
